# Supplementary material for: Biglycan Promotes Cancer Stem Cell Properties, NFκB Signaling and Metastatic Potential in Breast Cancer Cells
Source: Cancers (Basel). 2022 Jan 17;14(2):455. doi: 10.3390/cancers14020455 (PMC8773822; doi:10.3390/cancers14020455)

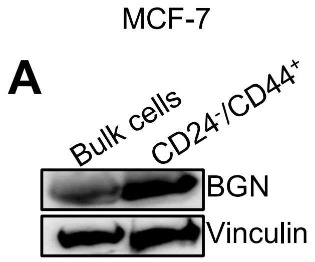

Figure S1: Upregulation of BGN in BCSCs derived from MCF-7. (A) Immunoblots depicting high BGN expression in sorted $CD24^{-}/CD44^{+}$ BCSCs from MCF-7 compared to bulk cancer cells.

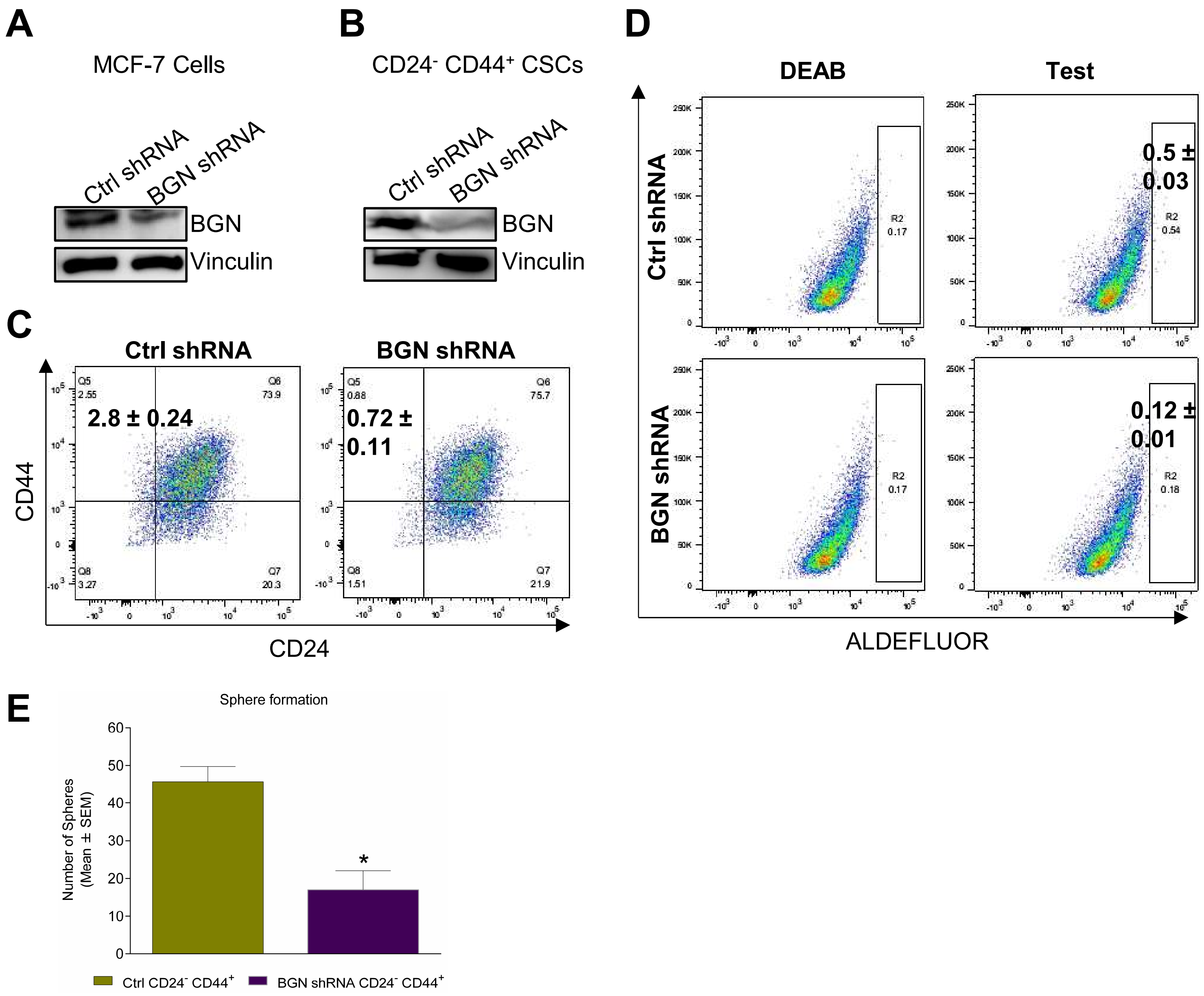

Figure S2: Loss of BGN in BCSCs exhibited reduction in CSC populations and sphere forming ability. (A) Immunoblot showing BGN expression in MCF-7 were stably expressing human BGN shRNA and Control shRNA. (B) Immunoblots depicting expression of BGN in $CD24^{-}/CD44^{+}$ BCSCs. (C) Dot plots of CD24 and CD44 marker expression analysis using FACS showing reduced number of $CD24^{-}/CD44^{+}$ BCSCs in BGN shRNA cells than Ctrl shRNA. (D) ALDH assay showing significant reduction of $ALDH^{+}$ CSCs with BGN knockdown. (E) Bar chart showing number of mammospheres formed by control shRNA $CD24^{-}/CD44^{+}$ and BGN shRNA $CD24^{-}/CD44^{+}$ BCSCs. Data are the mean ± SEM of three or four independent experiments. Statistical significance was determined using two-tailed *t* test; $*p \leq 0.05$ as compared to control shRNA. Number of spheres were quantified using ImageJ.

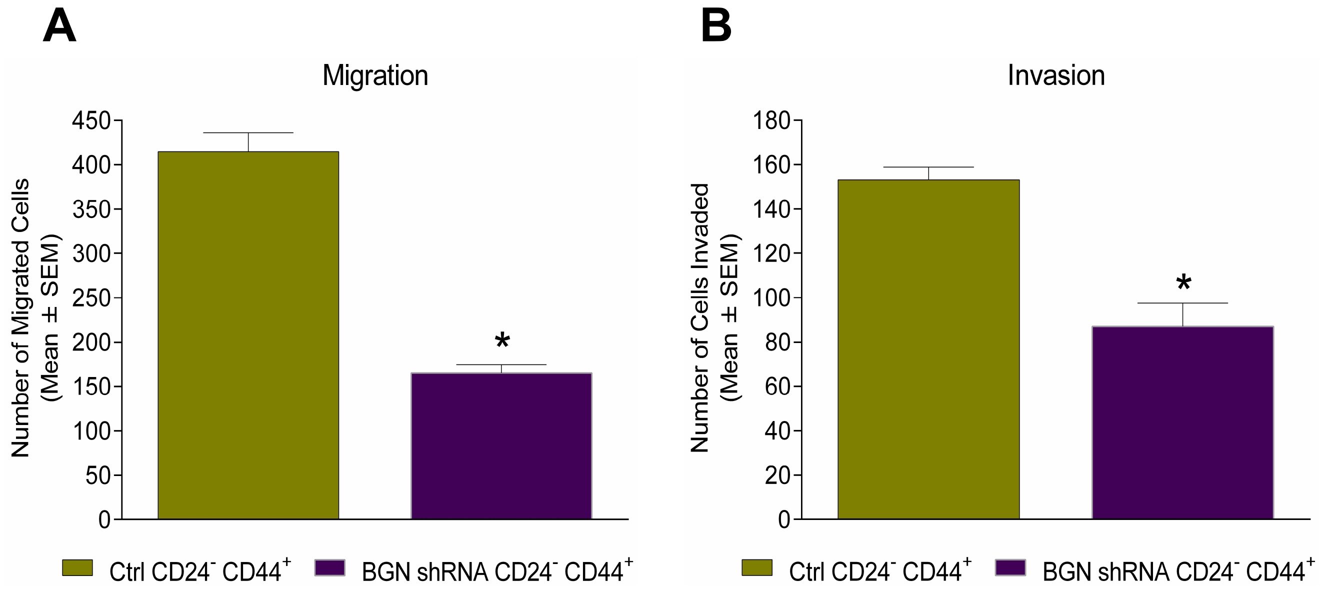

Figure S3: Loss of BGN reduces migration and invasion of BCSCs: (A) Bar charts showing a significant number of migrated cells were decreased at 24h in BGN shRNA $CD24^-/CD44^+$ CSCs. (B) Bar charts depicting a significant decreased number of invaded cells in BGN shRNA of $CD24^-/CD44^+$ CSCs. Data are the mean ± SEM of three independent experiments, $p \leq 0.05$ (t-test) as compared to *control shRNA group. Images of migration and invasion were quantified using ImageJ.

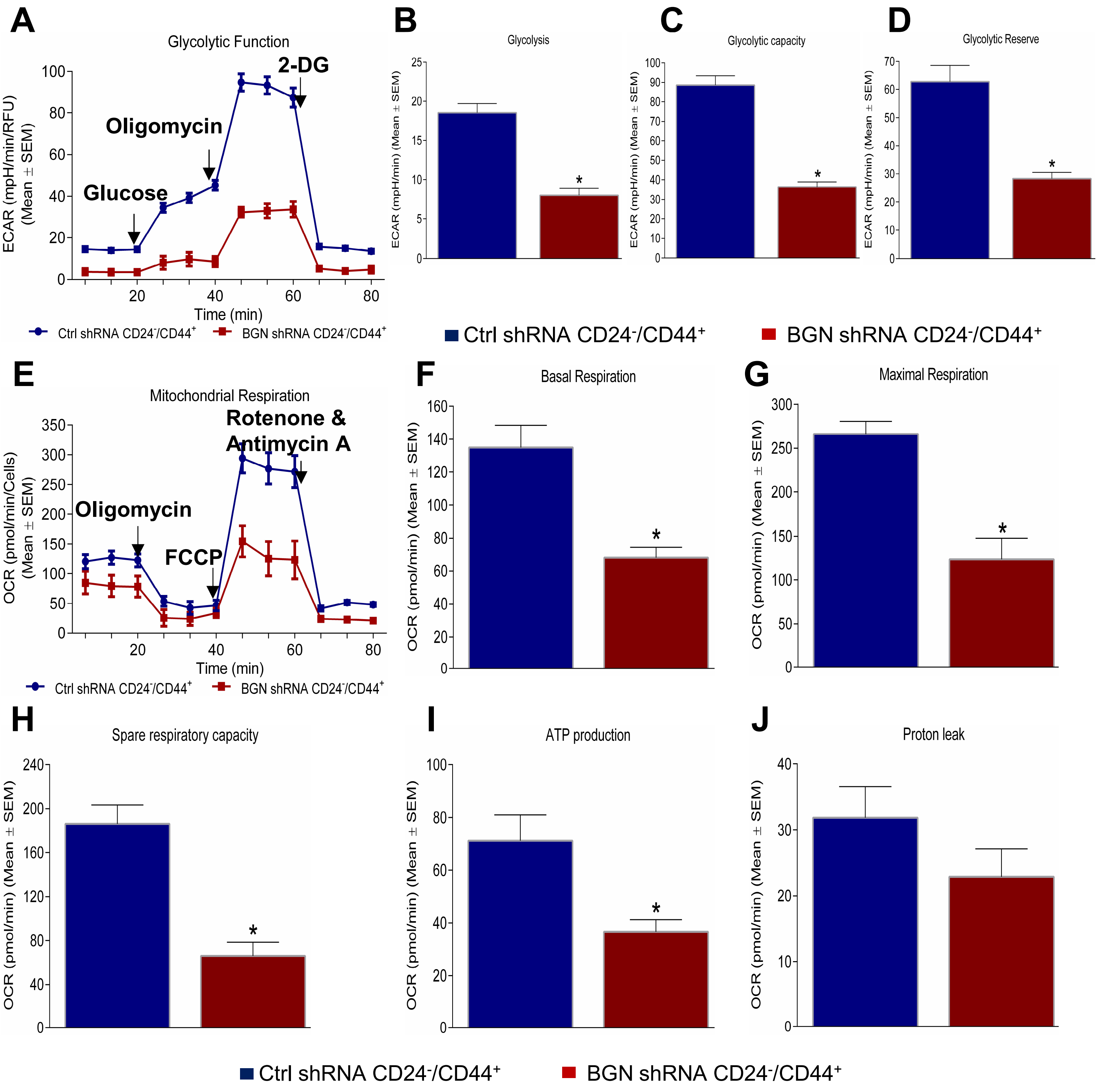

Figure S4: Role of BGN in metabolism of BCSCs: (A) A representative graph of ECAR outputs from the XF96 extracellular flux analyser. Bar charts showing the comparisons of (B) glycolysis, (C) glycolytic capacity, and (D) glycolytic reserve in control shRNA and BGN shRNA of BCSCs. (E) Representative graph of OCR outputs from the mitochondrial stress test of control shRNA and BGN shRNA of BCSCs in response to oligomycin, FCCP and rotenone and antimycin A. Bar charts displaying the assessments of (F) basal respiration, (G) maximal respiration, (H) spare respiratory capacity, (I) ATP production and (J) proton leak in control shRNA and BGN shRNA of BCSCs.

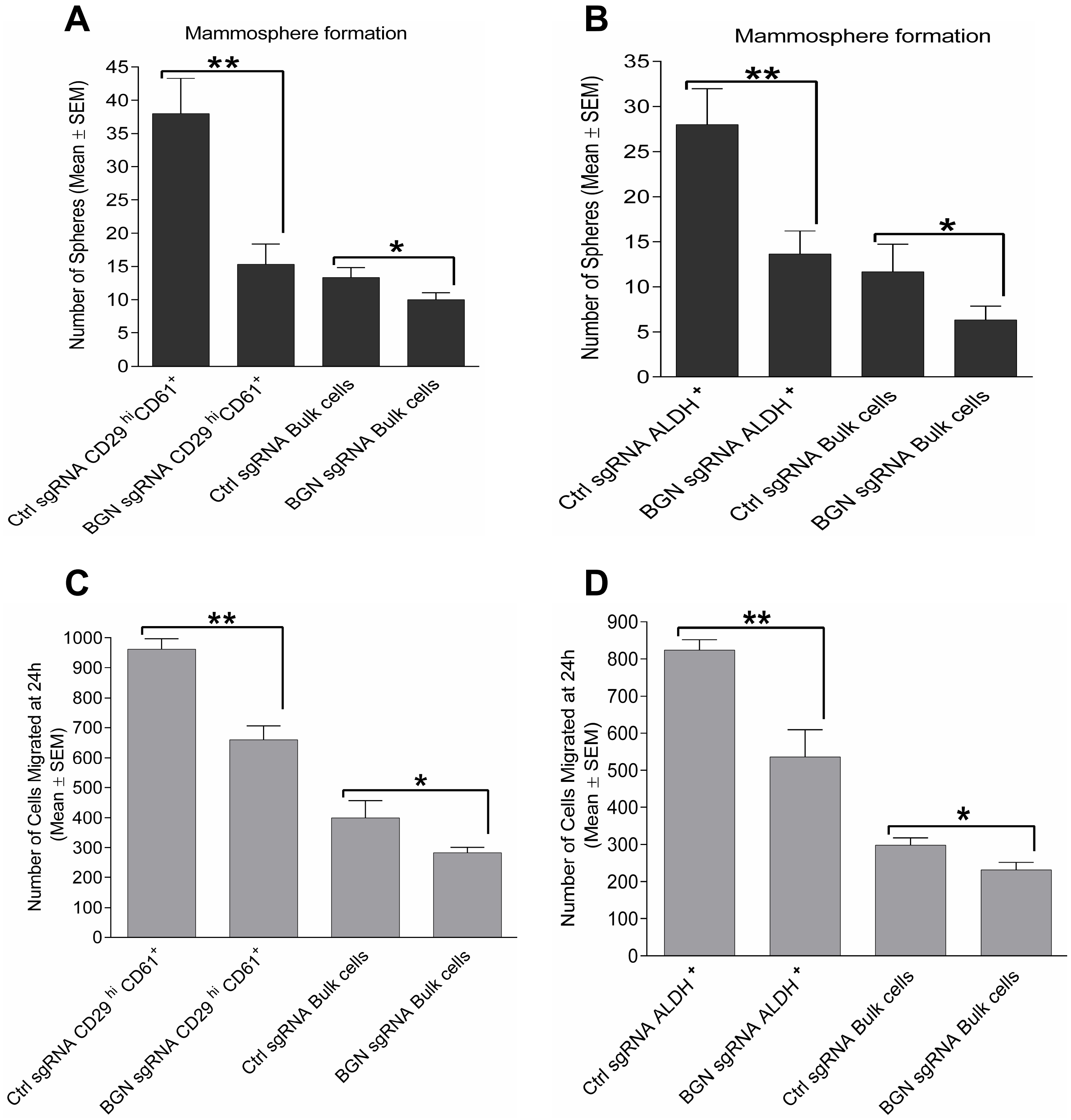

Figure S5: Loss of BGN reduces sphere formation and migration of BCSCs and bulk cells. (A, B) Bar chart showing number of mammospheres formed by control sgRNA and BGN sgRNA in BCSCs and bulk cells. Number of spheres were quantified using ImageJ. (C, D) Bar charts showing a significant number of migrated cells were decreased at 24h with depletion of BGN in BCSCs. Data are the mean ± SEM of three or four independent experiments. Statistical significance was determined using two-tailed *t* test; *$p \leq 0.05$ as compared to control sgRNA.

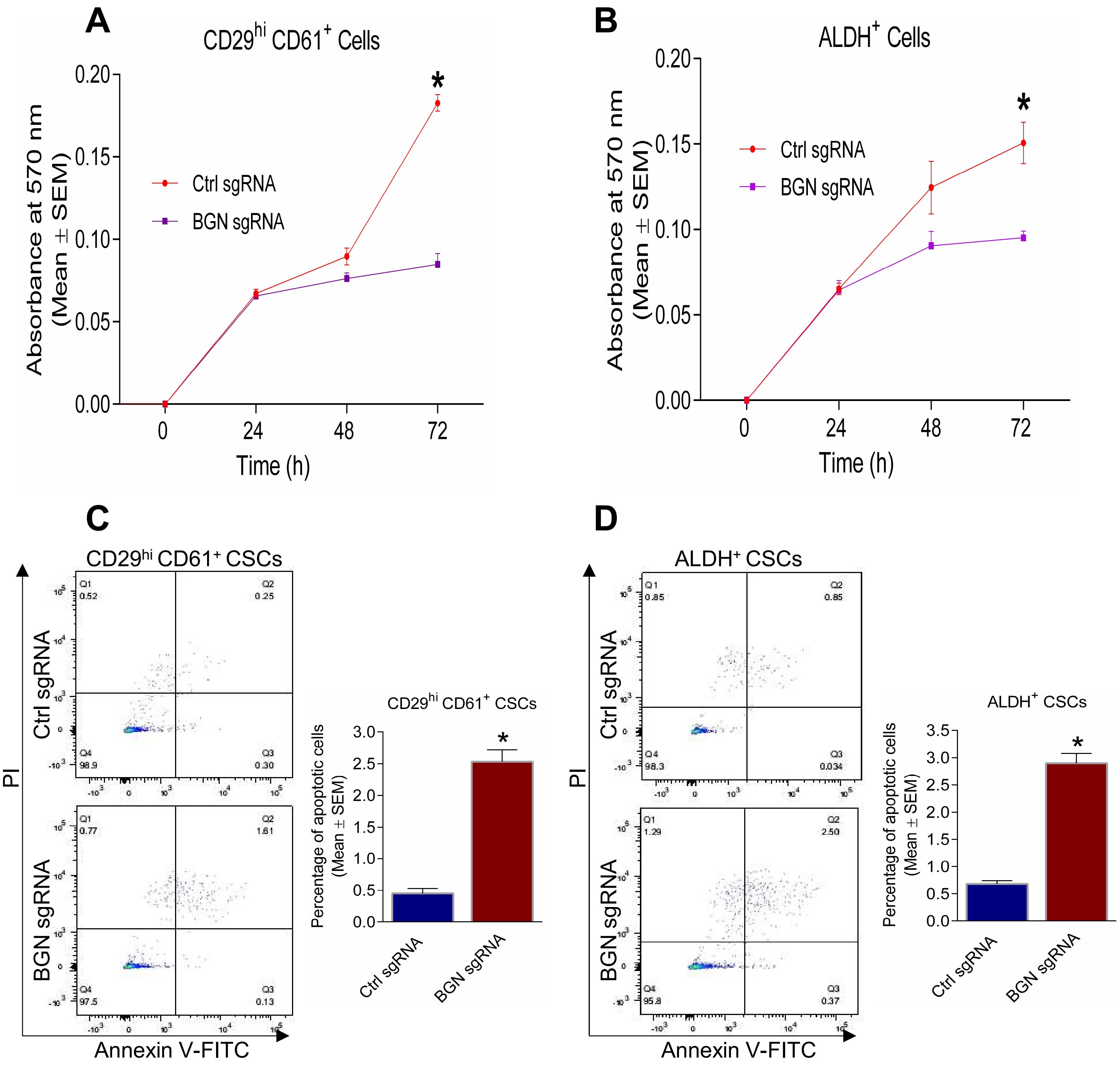

Figure S6: Loss of BGN reduces proliferation and induces apoptosis (A, B) Results of MTT assay depicting that depletion of BGN reduces proliferation of BCSCs at 72h. (C, D) Annexin-V FITC results showing that ablation of BGN induces apoptotic cells at later time points of 72h. Data are the mean ± SEM of three or four independent experiments. Statistical significance was determined using two-tailed *t* test; *p≤0.05 as compared to control sgRNA.

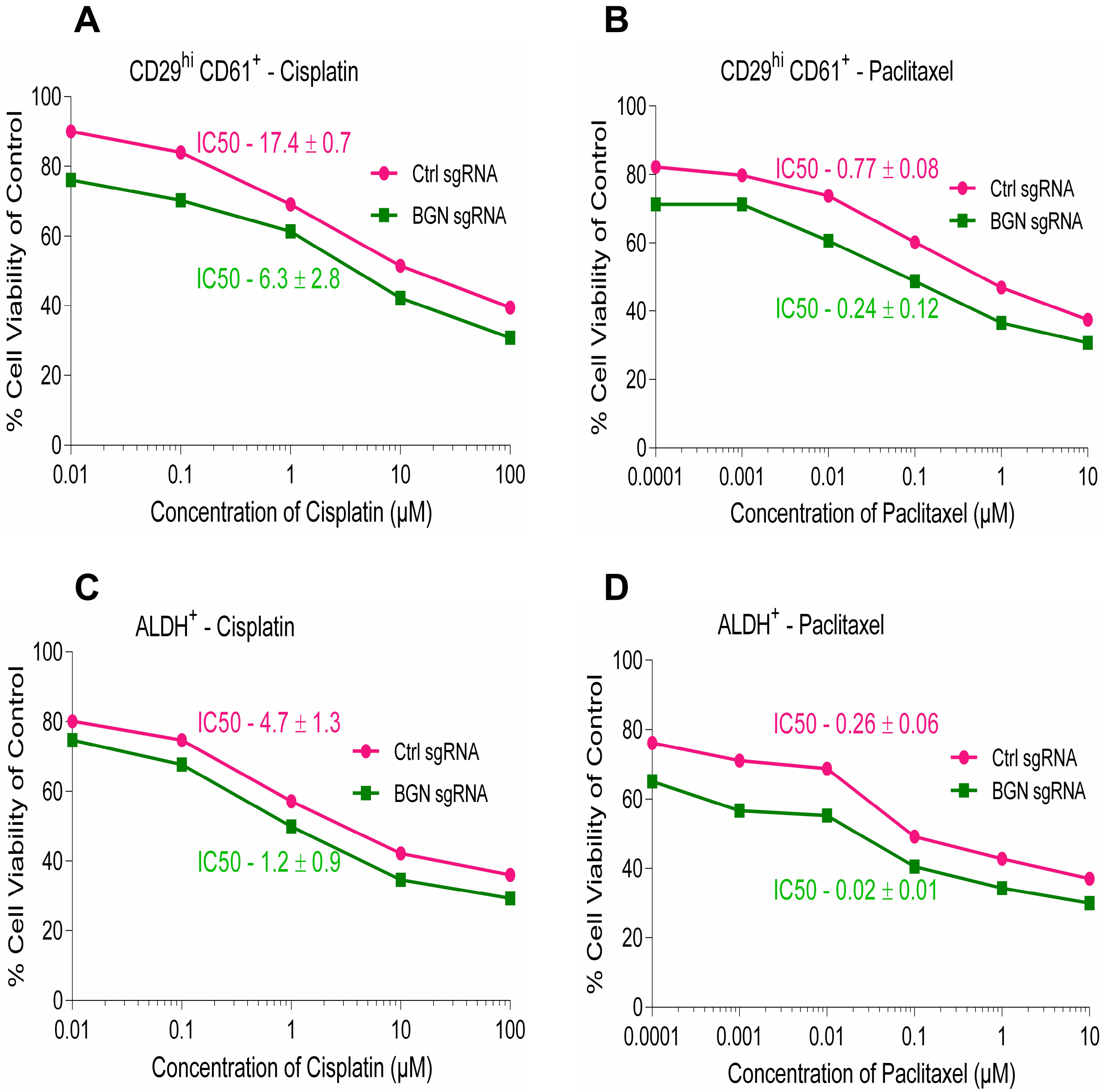

Figure S7: Loss of BGN enhanced chemosensitivity of BCSCs. (A, B) $CD29^{hi}$ $CD61^{+}$ BCSCs were treated with cisplatin and paclitaxel at different concentrations for 48h. and evaluated by MTT assay (C, D) $ALDH^{+}$ BCSCs with BGN depletion showed increased chemosensitivity in presence of cisplatin and paclitaxel. IC50 (mean ± SEM) measured from three independent experiments.

Supplementary Figure S8: Full size blots of Figure 1G, 1H and S1

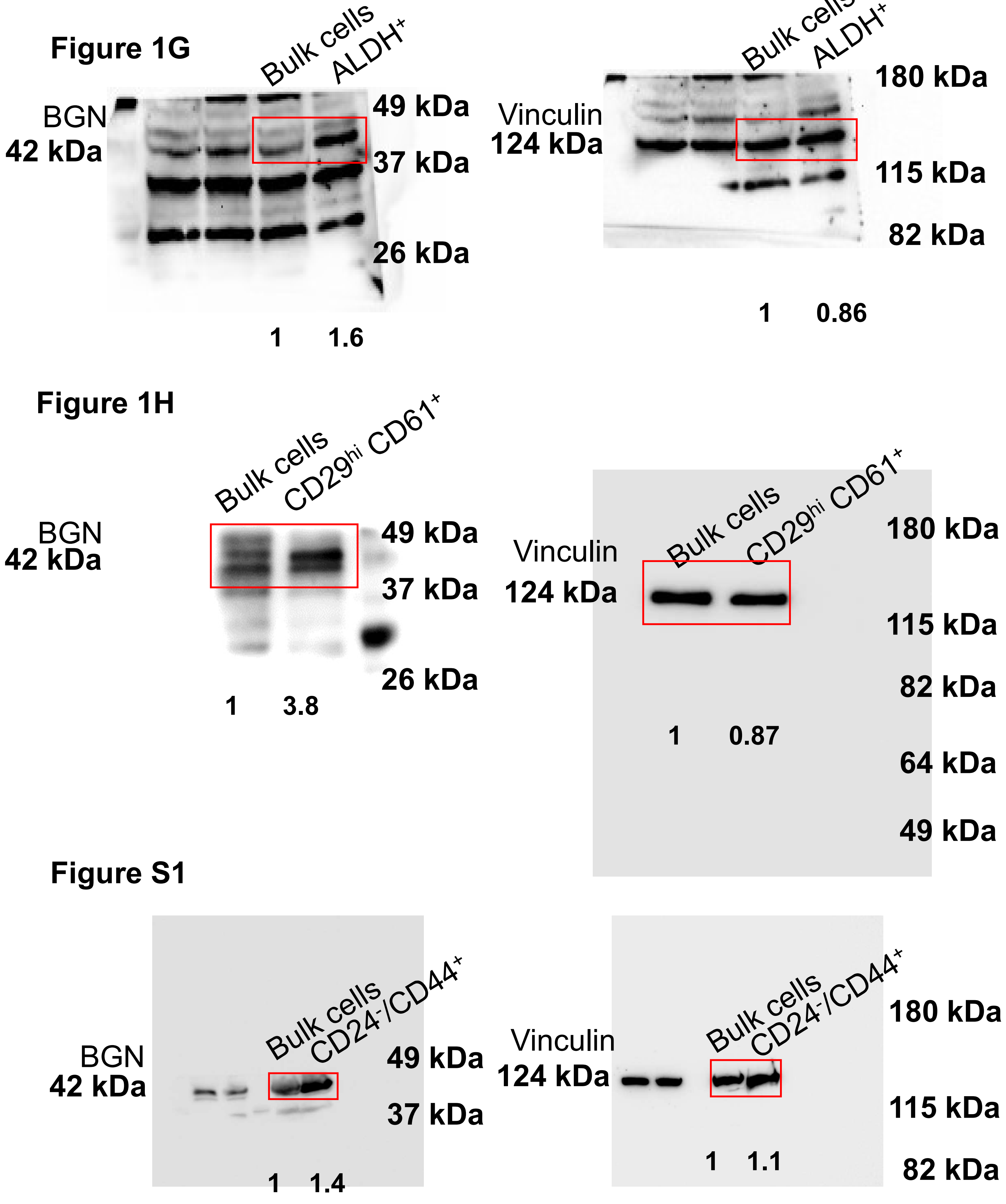

Supplementary Figure S9: Full size blots of Figure 2A, 2B and 2D

Figure 2A

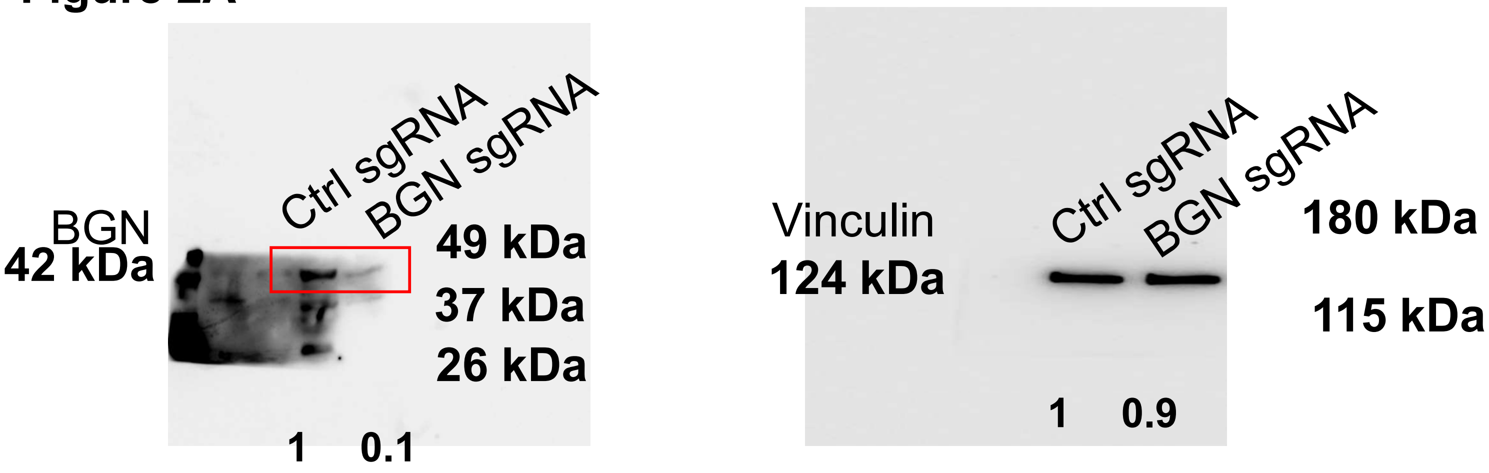

Figure 2B

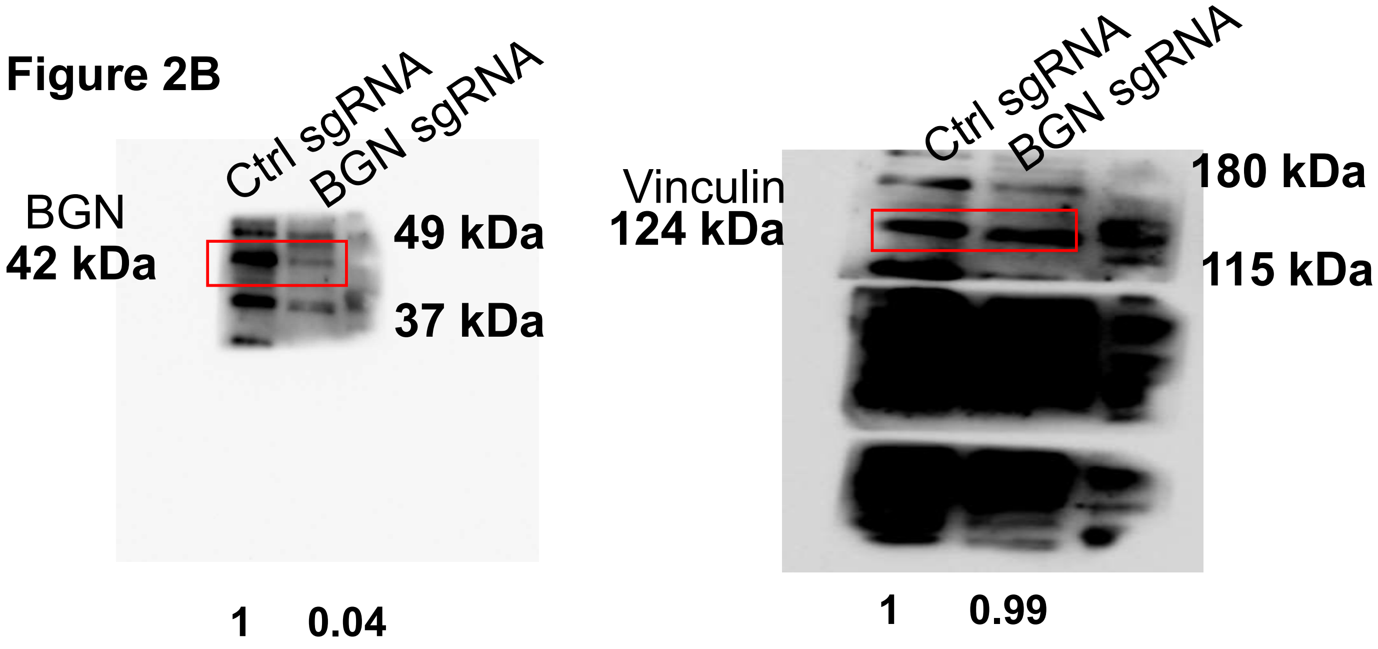

Figure 2D

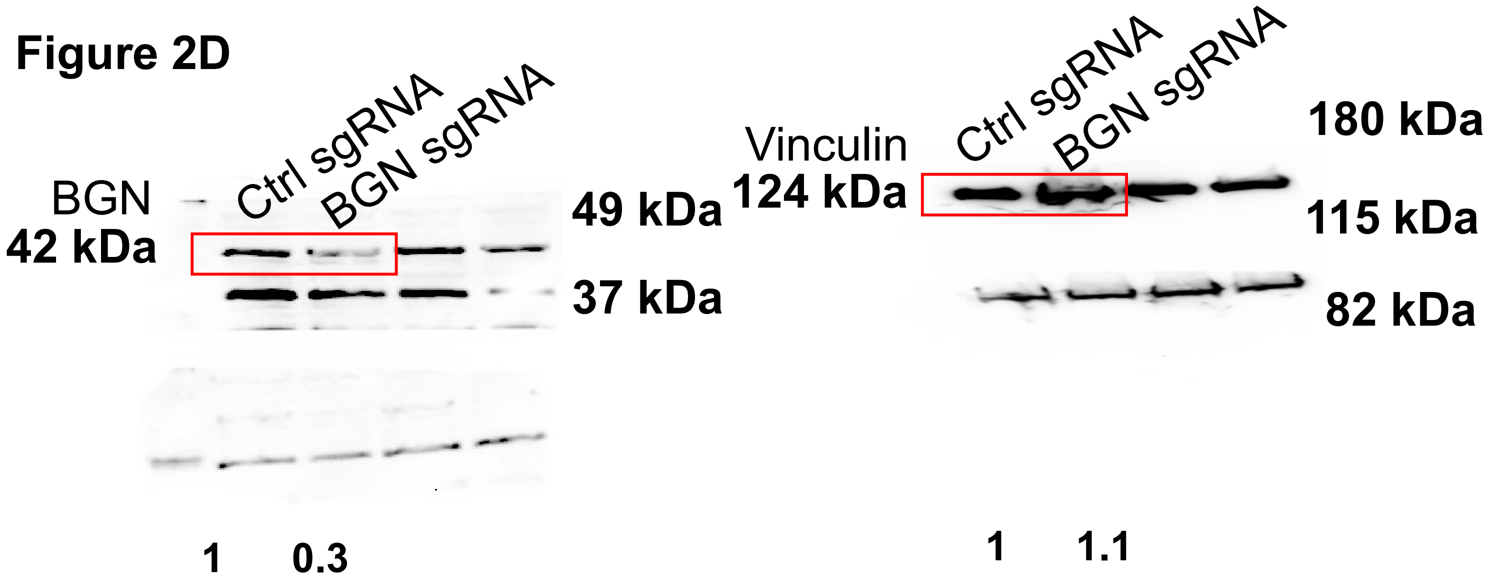

Supplementary Figure S10: Full size blots of Figure S2A and S2B

Figure S2A

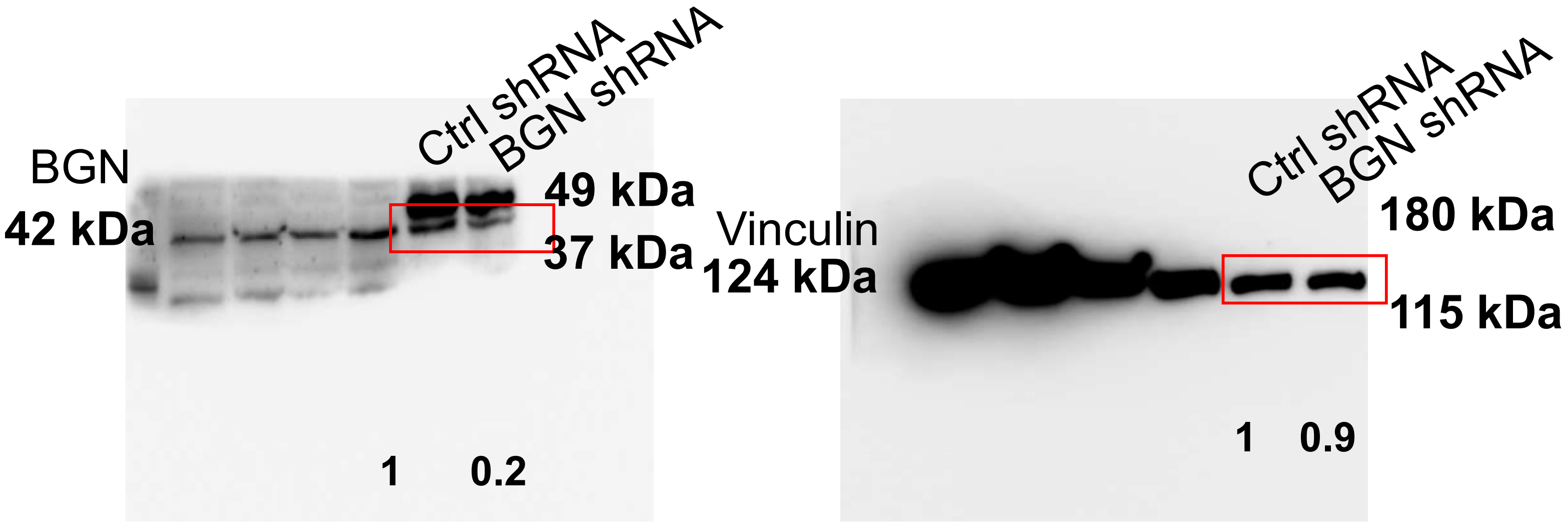

Figure S2B

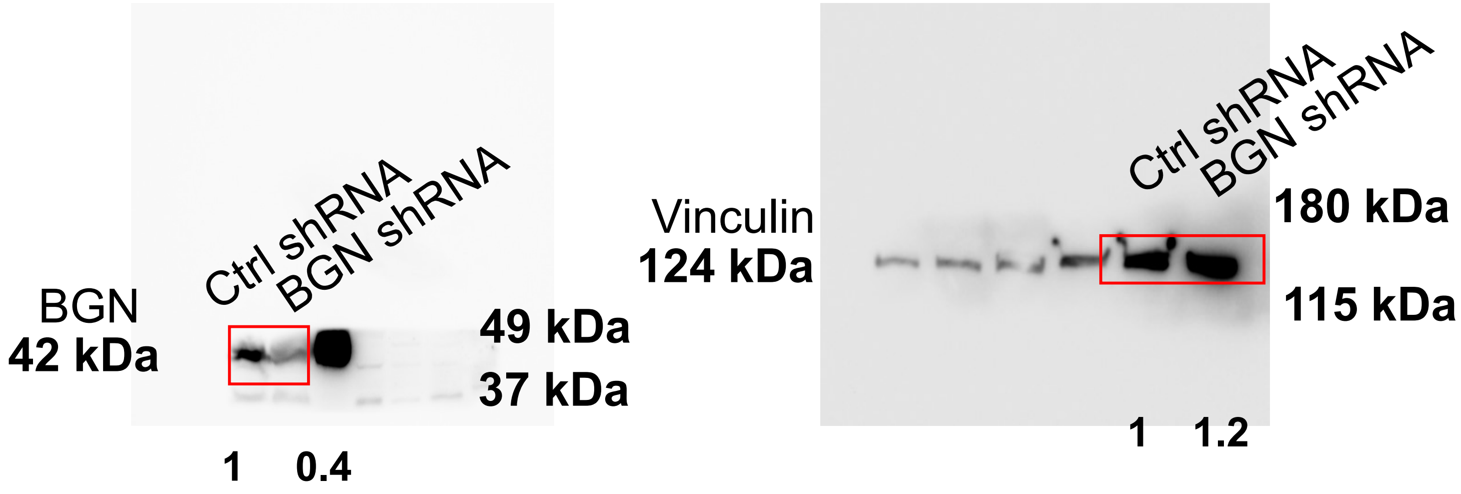

Supplementary Figure S11: Full size blots of Figure 5A

Figure 5A

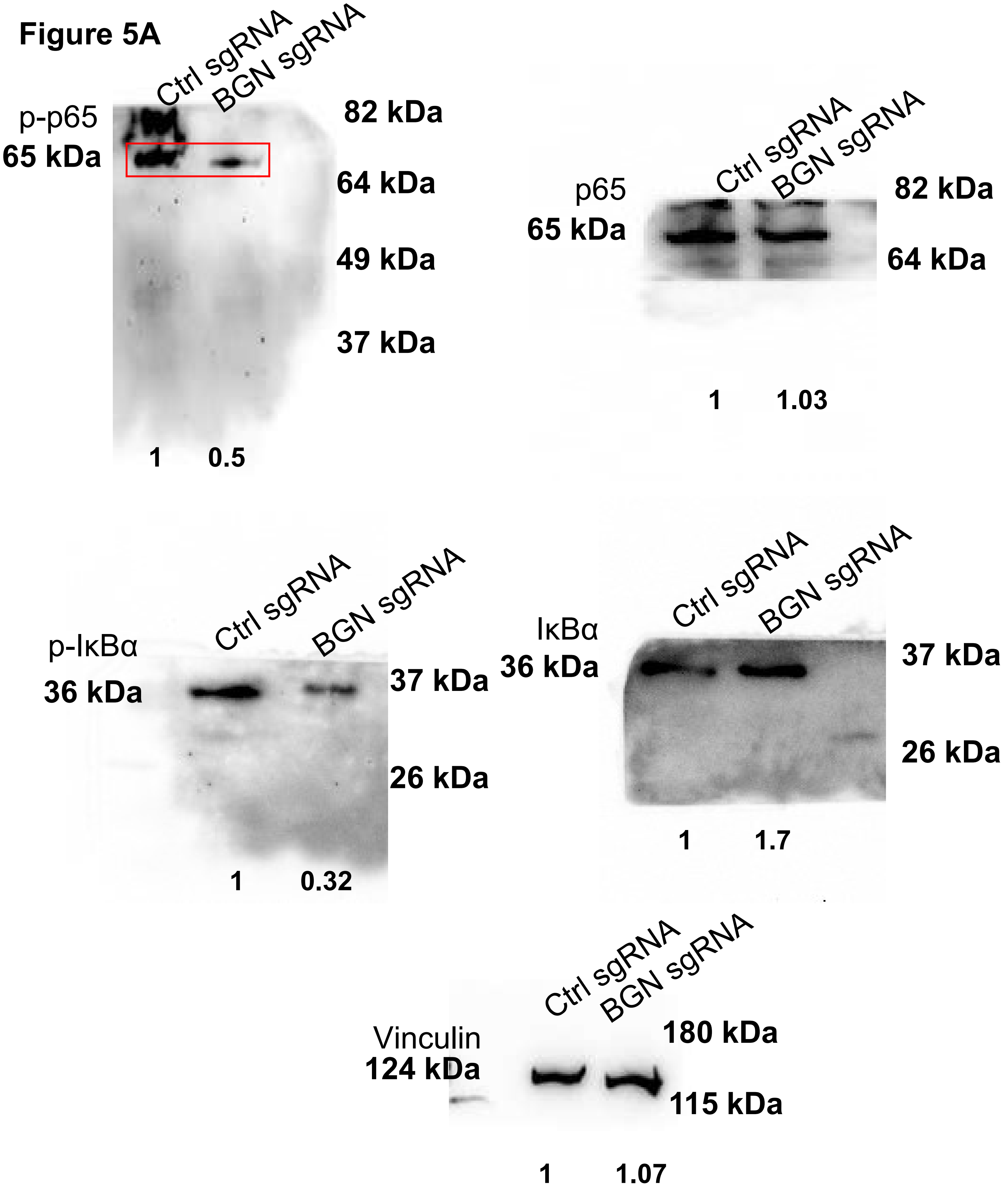

Supplementary Figure S12: Full size blots of Figure 5C

Figure 5C

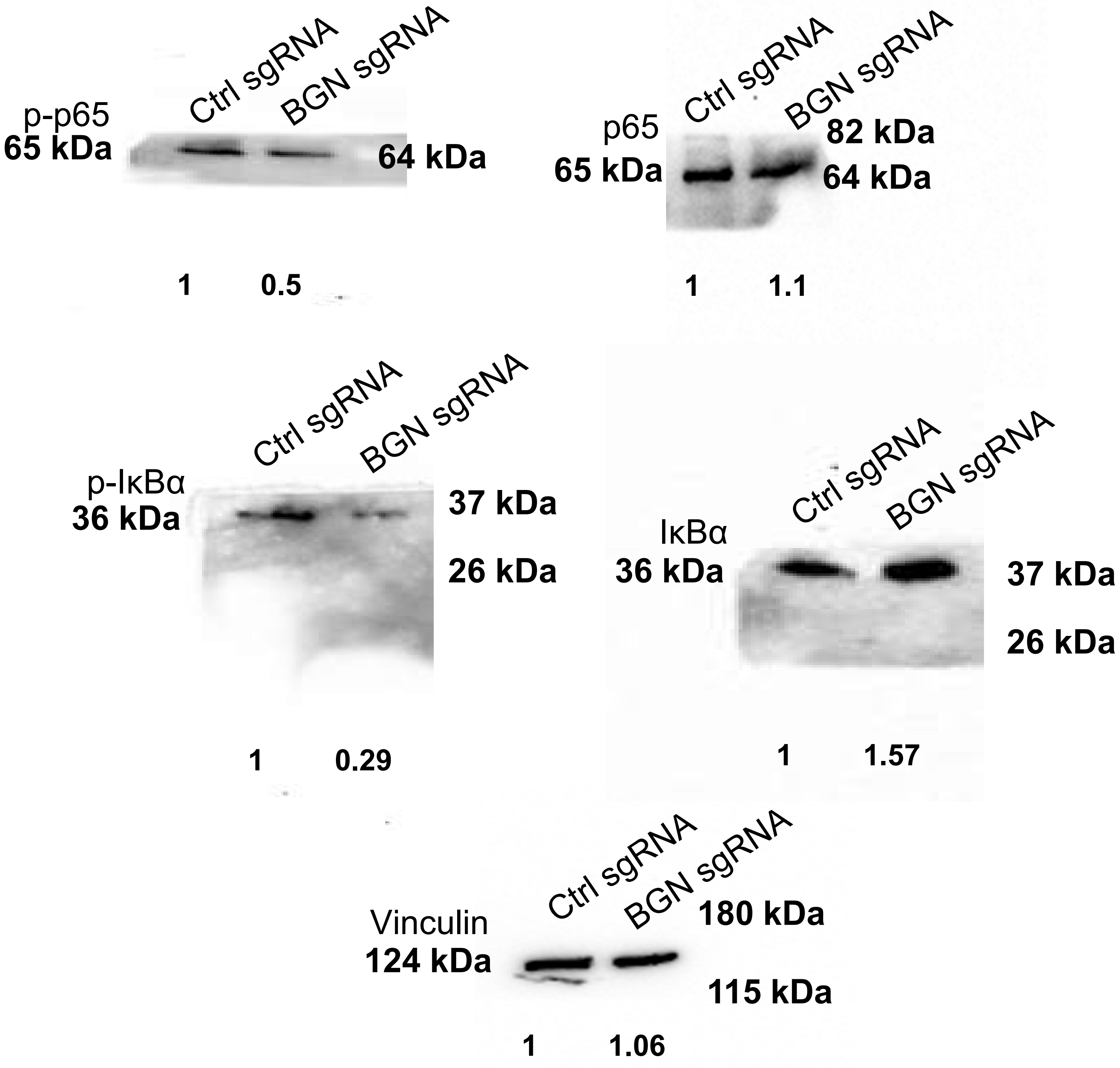

Supplement: Supplementary file 1 [file cancers-14-00455-s001.zip › cancers-1511658-supplementary.pdf]
